# Getting Bigger, Quicker? Gendered Socioeconomic Trajectories in Body Mass Index across the Adult Lifecourse: A Longitudinal Study of 21,403 Australians

**DOI:** 10.1371/journal.pone.0141499

**Published:** 2015-10-23

**Authors:** Xiaoqi Feng, Andrew Wilson

**Affiliations:** 1 Early Start Research Institute, University of Wollongong, Wollongong, Australia; 2 School of Health and Society, University of Wollongong, Wollongong, Australia; 3 Menzies Centre for Health Policy, University of Sydney, Sydney, Australia; 4 The Australian Prevention Partnership Centre, the Sax Institute, Sydney, Australia; University of Geneva, SWITZERLAND

## Abstract

Do socioeconomic inequities in body mass index (BMI) widen across the adult lifecourse? BMI data for 29,104 male and 32,454 female person-years aged 15 years and older (21,403 persons in total) were extracted from the Household, Income and Labour Dynamics in Australia between 2006 and 2012. Multilevel linear regression was used to examine age and gender specific trajectories in BMI by quintiles of neighborhood socioeconomic circumstance. Models were adjusted for probable sources of confounding, including couple status, number of children resident, if somebody in the household had been pregnant in the last 12 months, the highest level of education achieved, the average household gross income, and the percentage of time in the last year spent unemployed. Approximately 9.6% of BMI variation was observed between neighborhoods. High neighborhood disadvantage was associated with 2.09 kg/m^2^ heavier BMI (95%CI 1.82, 2.36). At age 15-24y, socioeconomic inequity in BMI was already evident among men and women especially (22.6 kg/m^2^ among women in the most affluent areas compared with 25.4 kg/m^2^ among the most disadvantaged). Among women only, the socioeconomic gap widened from 2.8 kg/m^2^ at age 15-24y to 3.2 kg/m^2^ by age 35-44y. Geographical factors may contribute to more rapid weight gain among women living in disadvantaged neighborhoods.

## Introduction

The high burden of obesity among adults in countries like the United States (US) and Australia poses daunting costs associated with cardiometabolic diseases like type 2 diabetes mellitus [1–5]. Though management of some cardiometabolic diseases can be effective [6], prevention–especially of obesity as a key determinant—ought to be the imperative for health policy.

A recent US report highlighted the need for better evidence on potential ‘obesogenic’ risk factors operating at the neighborhood scale [7]. Despite many scientific investigations published in this area [8], the identification of ‘neighborhood effects’ is well known to have many methodological challenges partially due to the reliance upon cross-sectional data [9]. A key strength of using longitudinal data is that change in body mass index (BMI) among participants living in more socioeconomically advantaged compared with disadvantaged neighborhoods can be potentially analyzed. However, not many longitudinal studies have been conducted in this regard and those which have report mixed findings [10–13]. Some have shown differential increases in BMI among participants living in more disadvantaged neighborhoods [10, 11], but others found no statistical evidence for this association [12, 13]. Moreover, we are aware of no study that has examined trajectories in BMI as people age in relation to neighborhood disadvantage and to what extent these differ by gender within the same analysis. This is an important gap in the scientific literature since BMI is not consistent across the adult lifecourse and gender differences are widely recognized [8, 14, 15], but identifying when socioeconomic trajectories in BMI diverge during adulthood is policy-relevant data for informing and enhancing prevention initiatives [16].

Two mainstream hypotheses support an overall theory of socioeconomic divergence (or widening socioeconomic inequity) in BMI across the lifecourse. Importantly, both of these hypotheses indicate that divergence occurs as a result of a more rapid weight gain among residents of socioeconomically disadvantaged neighborhoods relative to their counterparts in more affluent surroundings. The first hypothesis is ‘deprivation amplification’ [17], wherein socioeconomically disadvantaged neighborhoods are suggested to contain types of built environment that constrain healthy lifestyle choices and make less healthy options easier (e.g. an absence of stores selling fresh produce plus a high density of fast food takeaways in the local area privileging unhealthy dietary choices). Repeated exposure to these socioeconomically disadvantaged circumstances may result in more rapid weight gain as people age. The second hypothesis is ‘weathering' [18]; a psychosocial pathway that fits the classic accumulation hypothesis in lifecourse epidemiology [19]. This is a process of ‘wear and tear’ attributable to the clustering of negative experiences that accumulate differentially within disadvantaged neighborhoods, including discrimination (e.g. racial [20]), relative deprivation [21], violent crime [22], and disorder [23]. This psychosocial pathway is suggested to result in the overstimulation of allostatic systems and an increased propensity to gain weight [24–26].

These “deprivation amplification” and “weathering” hypotheses are probably very closely entwined, but it is not the aim of this paper to distinguish one from the other. Rather, the purpose of this paper is to investigate the broader hypothesis that residents of socioeconomically disadvantaged neighborhoods will not only be heavier than their counterparts in more affluent surroundings as younger adults, but that this inequity will widen as men and women age; potentially to differing degrees of magnitude by gender.

## Methods

### Design

No cohort data on weight status by age, gender and neighborhood socioeconomic circumstances was available for people across their entire adult lives. As an alternative, a growth curve methodology via an ‘accelerated longitudinal design’ was employed, which involved pooling groups of people of different ages repeatedly followed-up [27]. This design enabled the use of highly detailed panel data from the “Household, Income and Labour Dynamics in Australia’ (HILDA) [28]. Approval for the use of de-identified HILDA data was provided by the Government Department of Social Services.

### Data

We extracted 100,394 person-years of data from the 2006 to 2012 (inclusive) waves of HILDA. Access to HILDA was provided by the Melbourne Institute of Applied Economic and Social Research (University of Melbourne), with funding for data collection from the Australian Government through the Department of Social Services (DSS).

HILDA is an annual nationally representative panel survey that collects data on self-reported height and weight, from which body mass index (BMI) can be derived. Approximately 84,164 person years of data had valid BMI measures and a sub-set (n = 61,558) were residentially stable for at least 12 months prior to the survey. We focused on the residentially-stable subsample of person-years, which were from 18,341 participants aged 15 years and older (21,403 persons in total), nested within 5626 Census Collection Districts (CCDs). The rationale for focusing upon the subsample of residentially stable participants was our interest in neighborhood disadvantage as a predictor of BMI. The impact of one on the other is unlikely to occur within one year, with contextual influences on health status like BMI generally hypothesized to occur over a longer period [29]. The mean number of person-year observations per participant was 2.9 (ranging from 1 to 6), with 3759 of 21,403 participants observed in one year only. The gender distribution of person-years was 29,104 for men and 32,454 for women. BMI averaged at 26.7 with a standard deviation of 5.6. The main outcome variable was BMI, which was normally distributed in continuous format.

The CCD of residence for every participant was linked by the data provider to the area-level measure of socioeconomic circumstances: the Socio Economic Index For Areas (SEIFA) index. We used the index of relative advantage/disadvantage, which summarizes multiple Census variables that describe positive and negative aspects of socioeconomic circumstances [30].

A range of variables were identified to help address probable sources of confounding based upon a synthesis of previous literature [8, 29, 31]. These included gender, age (linear and square terms), demographic and person-level socioeconomic factors. Demographic factors consisted of whether a participant was living on their own or as part of a couple (married or cohabiting), the number of children in the household (0, 1, 2, 3 or more) and if somebody in the household had been pregnant in the last 12 months (not the participant specifically). Socioeconomic confounders included the highest level of education achieved (less than high school, high school to advanced diploma, university or higher), average household gross income (expressed in quintiles) and the percentage of time in the last year spent unemployed.

### Statistical analysis

A multilevel framework was used to model annually repeated measures of BMI at level 1, within participants at level 2, nested within CCDs at level 3. This multilevel linear regression was estimated in MLwIN 2.30 [32], with the ‘Variance Partition Coefficient’ (VPC) used to describe the relative contributions of phenomena operating at each level to the overall variation in BMI among the sample. In Model 1, gender-specific growth curves were fitted with gender and age linear and square variables and 2-way interaction terms. Model 2 introduced dummy variables for neighborhood socioeconomic quintiles. Socioeconomic and other demographic confounders were added in Model 3. Finally, Model 4 introduced a three-way interaction term to investigate BMI trajectories by age and gender across different quintiles of neighborhood disadvantage. Predicted mean adjusted growth curves were illustrated to afford interpretation of the 3-way interaction term.

## Results

Of the 61,558 person-years, 39.7% were within the normal range (18.5 to 24.9 kg/m^2^), 34.8% were overweight (25.0 to 29.9 kg/m^2^), 22.9% classified as obese (≥ 30.0 kg/m^2^) and 2.6% considered underweight (<18.5 kg/m^2^). The average age was 46.6 with a standard deviation of 18.5, a minimum of 18 and a maximum of 100. A total of 64.7% were living as part of a couple (legally married or de facto), 32.8% had less than high school education and 87.9% reported zero time spent unemployed in the last 12 months. The average household gross income was AU$106,490 with a standard deviation of AU$102,690. A total of 4.7% of the sample reported a pregnancy within the household in the last 12 months and 62.5% had no children.

The age- and gender-adjusted multilevel model (Table 1, Model 1) showed that approximately 73% of the variation in BMI was observed between participants, around 17.8% over time and 9.6% between CCDs. Males tended to have higher BMI than females on average, particularly at younger ages. Higher BMI was associated with higher levels of neighborhood socioeconomic disadvantage (e.g. quintile 5 coefficient 2.09, 95% confidence interval (95%CI 1.82, 2.36)). Adjustment for confounding variables made negligible impact on the association between BMI and neighborhood socioeconomic disadvantage (Model 3). BMI was higher among participants living as part of a couple, those who spent 75% or longer of the last 12 months unemployed, those with a mid-level of education (high school to advanced diploma), and those with a pregnancy in the household in the last 12 months. No significant associations were observed for household gross income or the number of children in the household.

**Table 1:** Multilevel models.

	Model 1	Model 2	Model 3
**Fixed part**	Coefficient (95% Confidence Interval)		
Grand mean intercept	18.243 (17.114, 19.372)	16.986 (15.846, 18.126)	17.722 (16.561, 18.884)
Gender (ref: Male)			
Female	0.118 (-0.582, 0.818)	0.095 (-0.604, 0.795)	-0.126 (-0.830, 0.578)
Age	0.413 (0.360, 0.465)	0.416 (0.364, 0.468)	0.394 (0.341, 0.447)
Age^2^	-0.004 (-0.005, -0.003)	-0.004 (-0.005, -0.003)	-0.004 (-0.004, -0.003)
Gender*Age	-0.044 (-0.076, -0.012)	-0.044 (-0.076, -0.012)	-0.034 (-0.067, -0.001)
Gender*Age^2^	0.001 (0.000, 0.001)	0.001 (0.000, 0.001)	0.001 (0.000, 0.001)
Neighbourhood disadvantage (ref: quintile 1)			
quintile 2		0.904 (0.631, 1.176)	0.820 (0.548, 1.091)
quintile 3		1.501 (1.222, 1.779)	1.361 (1.082, 1.641)
quintile 4		1.666 (1.391, 1.941)	1.510 (1.232, 1.788)
quintile 5		2.152 (1.880, 2.424)	2.003 (1.726, 2.280)
Couple status (ref: in a couple)			
not in a couple			-0.348 (-0.473, -0.223)
refused			-0.388 (-1.394, 0.618)
% time unemployed			
1–24%			-0.082 (-0.230, 0.065)
25–49%			-0.090 (-0.286, 0.106)
50–74%			0.122 (-0.121, 0.365)
75–100%			0.290 (0.083, 0.497)
not asked			-0.092 (-0.219, 0.036)
Highest educational qualification (ref: <year 12)			
year 12 to adv. diploma%			0.192 (0.069, 0.316)
university			-0.520 (-0.704, -0.335)
undetermined			-1.384 (-4.870, 2.103)
Annual household disposable income (ref: quintile 1)			
quintile 2			-0.013 (-0.107, 0.082)
ਁ quintile 3			-0.019 (-0.127, 0.090)
quintile 4			0.019 (-0.097, 0.135)
quintile 5 (high)			0.059 (-0.066, 0.184)
Pregnancy in the last 12 months? (ref: no)			
yes			0.777 (0.599, 0.954)
Missing/Refused			-0.229 (-0.553, 0.095)
Number of children in household (ref: 0)			
1			-0.019 (-0.170, 0.133)
2			-0.293 (-0.478, -0.107)
>3			-0.038 (-0.292, 0.216)
**Random part**			
CCD (N = 5626)			
Variance (95%CI)	3.01 (2.62, 3.40)	2.24 (1.90, 2.58)	2.13 (1.79, 2.46)
VPC	9.9%	7.5%	7.2%
Person (N = 21403)			
Variance (95%CI)	21.95 (21.43, 22.46)	22.07 (21.56, 22.59)	22.02 (21.51, 22.54)
VPC	72.3%	74.3%	74.6%
Observation (N = 61558)			
Variance (95%CI)	5.38 (5.31, 5.46)	5.38 (5.31, 5.46)	5.37 (5.29, 5.44)
VPC	17.7%	18.1%	18.2%
loglikelihood	-165328.75	-165194.39	-165089.58

CCD: Census Collection District | VPC: Variance Partition Coefficient | 95%CI: 95% Confidence Intervals

**Fig 1:**
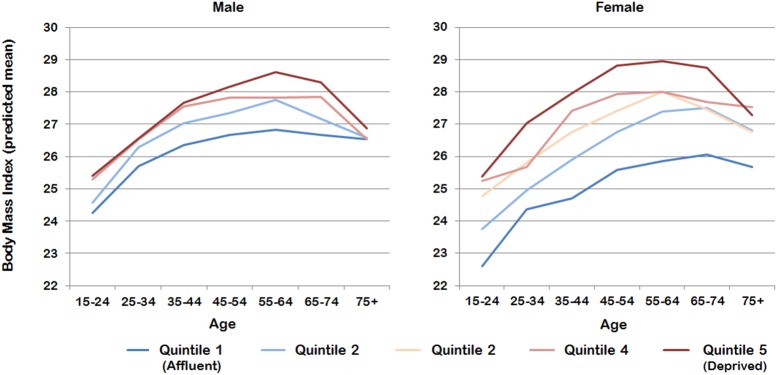
Socioeconomic trajectories in body mass index (BMI) across the adult lifecourse: adjusted mean BMI from a multilevel model with a 3-way interaction between neighborhood deprivation, age and gender

Fig 1 illustrates the adjusted BMI growth curves predicted from an extension of Model 3 using a three-way interaction between age, gender and neighborhood socioeconomic disadvantage. At age 15-24y, socioeconomic inequity in BMI was already evident among men and, especially women (22.6 kg/m^2^ among the most affluent areas compared with 25.4 kg/m^2^ among the most disadvantaged). For men, this gap remained reasonably constant throughout adulthood until a socioeconomic convergence from 75 years and older. Among women, the socioeconomic gap widened from 2.8 kg/m^2^ at age 15-24y to 3.2 kg/m^2^ by age 35-44y. Within the most affluent areas, the gender gap narrowed by age, with males 1.7 kg/m^2^ higher than women at age 15-24y, converging to just 0.6 kg/m^2^ by age 65-74y. In contrast, there was marginal gender difference at age 15-24y between men and women in the most disadvantaged areas, but this rose to 0.7 kg/m^2^ higher among women by age 45-54y.

## Discussion

Many studies report higher BMI among the residents of socioeconomically disadvantaged areas [8, 14, 15], though not all [33]. This study provides support for the mainstream finding. More importantly, it also reports new evidence that reveals an inconsistency in the degree of association between BMI and neighborhood socioeconomic circumstances by age and gender. For men and especially women, socioeconomic inequity in BMI observed among younger adults widens as people age. This is due to a faster gain in BMI among people living in more disadvantaged neighborhoods relative to their counterparts in more affluent areas. To illustrate this pattern, the mean BMI among men and women in the most disadvantaged neighborhoods at age 15-24y was 25.4 kg/m^2^. Men in the most affluent areas only reached this BMI level by age 25-34y, while women in the most advantaged surroundings similarly only achieved a comparable BMI by age 45-54y. This indicates that above and beyond demographic and personal socioeconomic circumstances, people living in more disadvantaged areas not only experience higher BMI from younger adulthood, but for women in particular, this inequity also widens across the adult lifecourse.

This is not to say that neighborhood disadvantage is the definitive causal agent of the widening socioeconomic inequity in BMI across the lifecourse. Potentially, the level of neighborhood disadvantage is a proxy for other spatially manifesting factors that influence BMI via deprivation amplification and/or weathering pathways. Distinguishing between these pathways was not the aim of this study, but it should be noted that our results are nonetheless based upon observational longitudinal data that is prone to a range of methodological problems, notably but not limited to confounding. We controlled for several individual-level characteristics in order to adjust for systematic differences between people living in neighborhoods of contrasting socioeconomic circumstances, but there may be other unmeasured factors that influence both where people live and their weight. Early life experiences and prior health could be potentially important factors, since it is well known that neighborhood relocation is health selective [34–37].

An important part of the identification challenge is that people do not choose their neighborhoods of residence by flip of a coin and, for some commentators, once all individual-level confounders are taken into account, there may be little variation in neighborhood socioeconomic circumstances left to ‘explain’ [9]. This daunting prospect is likely to depend upon context, however, with recent evidence from a study of HILDA showing significant ‘off-diagonal’ residential mobility in Australia [38]. The ‘off-diagonal’ aspect refers to the extent that people move to a new area that is substantively different in some shape or form to the previous neighborhood of residence–socioeconomic disadvantage in the case of the previous Australian study. The identification of causal neighborhood effects will also depend upon time, with factors that influence where people live and potential weight gain unlikely to be consistent across the lifecourse nor across generations and, furthermore, unlikely to result in instantaneous impacts on BMI (though influences on other health status such as mental wellbeing may be more proximal). The bleak outlook for distinguishing between age-period-cohort effects is increasingly appreciated [39–41] and the question of how to conceptualize and measure the temporal dynamics of neighborhood effects on health remains a ‘work in progress’ for the discipline of Social Epidemiology more generally. The issue of outcome misclassification due to long-known differences in the self-reporting of height and weight between genders and age groups is another limitation that needs to be acknowledged [42]. These challenges apply to the pooled longitudinal design that we have used, though the availability of a cohort study tracking people fully across their lives at regular time intervals and with consistently measured indicators would still be subject to age-period-cohort limitations.

In conclusion, the observations in this study indicate that women living in socioeconomically disadvantaged neighborhoods not only have heavier BMIs in young adulthood, but the rate of weight gain appears to be potentially higher as they age. This means that socioeconomic inequity in BMI among women tends to widen across adulthood. This pattern was not as obvious for men. Future work needs to focus on the identification of ‘causal’ effects of neighborhood socioeconomic circumstances on weight gain. Second, it would be operative for that work to attempt to differentiate between factors attributable to deprivation amplification (e.g. mediation via behaviors shaped by material conditions) and weathering (e.g. mediation by psychosocial risk factors) hypotheses. Given the likelihood that these studies will continue to rely upon observational data, inferences must remain conservative but relatively new techniques, such as causal mediation models (or ‘marginal structural models’) could provide new insights [43, 44]. Further steps may also include studies that attempt to imitate randomized trials by focusing upon within-person change in BMI across different age groups and by gender, with the putative interventions mimicked either by a change in contextual exposure due to neighborhood relocation [36, 37, 45], or as a result of the surroundings changing while the study participants remain within the same places [46].
